# Ten-year outcomes after DMEK, DSAEK, and PK: insights on graft survival, endothelial cell density loss, rejection and visual acuity

**DOI:** 10.1038/s41598-025-85138-4

**Published:** 2025-01-08

**Authors:** Theresa Isabelle Wilhelm, Laura Gauché, Daniel Böhringer, Philip Maier, Sonja Heinzelmann, Mateusz Glegola, Paola Kammrath Betancor, Thomas Reinhard

**Affiliations:** Eye Center, Medical Center, Faculty of Medicine, University of Freiburg, Kilianstraße 5, 79106 Freiburg, Germany

**Keywords:** DMEK, DSAEK, Penetrating keratoplasty, Corneal transplantation, Fuchs endothelial corneal dystrophy, Graft survival, Clinical trials, Outcomes research

## Abstract

Fuchs Endothelial Corneal Dystrophy (FECD) is the most frequent indication for corneal transplantation, with Descemet membrane endothelial keratoplasty (DMEK), Descemet stripping automated endothelial keratoplasty (DSAEK), and penetrating keratoplasty (PK) being viable options. This retrospective study compared 10-year outcomes of these techniques in a large cohort of 2956 first-time keratoplasty eyes treated for FECD at a high-volume corneal transplant center in Germany. While DMEK and DSAEK provided faster visual recovery (median time to BSCVA ≥ 6/12 Snellen: DMEK 7.8 months, DSAEK 12.4 months, PK 37.9 months; cumulative probability of BSCVA ≥ 6/12 Snellen within 5 years: DMEK 93%, DSAEK 83%, PK 63%), PK surprisingly exhibited superior long-term graft survival (92% vs. 75% for DMEK and 73% for DSAEK at 10 years). Endothelial cell density (ECD) decreased significantly faster after DMEK and DSAEK, potentially contributing to their lower graft survival (10-year ECD > 1000 cells/mm^2^ probability: DMEK 3%, DSAEK 8%, PK 18%). DMEK demonstrated the lowest rejection rate (10% at 10 years vs. 13% for PK and 19% for DSAEK). These findings challenge the perceived superiority of endothelial keratoplasty and highlight the need for further investigation into the long-term implications of accelerated endothelial cell loss after DMEK and DSAEK.

## Introduction

Fuchs endothelial corneal dystrophy (FECD) is a progressive disease characterized by accelerated loss of corneal endothelial cells, leading to corneal edema and visual impairment^1^. For patients with advanced FECD, surgical replacement of the diseased endothelium via corneal transplantation is the primary treatment option, making it the leading indication for corneal transplantation worldwide^2^. Historically, the gold standard procedure was penetrating keratoplasty (PK), which involves full-thickness replacement of the patient’s cornea with donor tissue. However, in the past two decades, selective endothelial keratoplasty techniques like Descemet membrane endothelial keratoplasty (DMEK), first described by Melles et al., and Descemet stripping automated endothelial keratoplasty (DSAEK) have emerged as preferred alternatives. These procedures selectively replace only the diseased endothelial layer, leaving most of the patient’s cornea intact^3–6^.

These lamellar techniques have been reported to offer faster visual recovery, better visual outcomes, and lower graft rejection rates than PK, potentially shortening long-term corticosteroid therapy and thus reducing the risk of steroid response^5^. Despite the clinical enthusiasm for DMEK and DSAEK, deriving from their purportedly superior outcomes, the literature presents a mixed picture regarding long-term outcomes. Early reports have been encouraging, suggesting excellent clinical outcomes with these lamellar techniques^7–9^. However, contradictory findings from large national keratoplasty registries in Australia and the United Kingdom, particularly with regard to graft survival and endothelial cell loss, require further investigation^10,11^.

This retrospective study aims to provide real-world comparative data on the 10-year outcomes following DMEK, DSAEK, and PK for patients undergoing surgery for FECD. By analyzing a large cohort of patients who underwent corneal transplantation at a high-volume corneal transplant center in Germany for the same indication, we seek to evaluate the long-term graft survival, endothelial cell loss, rejection rates, and visual recovery associated with each surgical technique. The findings from this study can offer valuable insights to ophthalmologists and patients regarding the potential risks and benefits of the different transplantation approaches for treating advanced FECD.

## Methods

This retrospective, observational study aimed to compare the 10-year outcomes following DMEK, DSAEK, PK. The study was conducted at a single center, the Eye Center of the Albert-Ludwig-University Hospital Freiburg, using a comprehensive database containing the eye center medical records and the Lions Cornea Bank Baden-Wuerttemberg data. The study cohort included all first-time keratoplasty eyes treated for FECD consecutively at the Eye Center from November 2003 up to and including October 2023.

All grafts were stored under organ culture conditions following national and European guidelines. Standard DMEK, DSAEK, and PK techniques were used and performed under general anesthesia, as described earlier^12^. All surgeons followed standardized surgical protocols for each technique that was not significantly changed over time. Briefly, PK was performed using mechanical trephination and double running sutures. A guided trephine system (Polytech, Germany, diameter 8 mm) was used for PK. DSAEK was performed using a Busin glide and grafts ranging between 80 and 150 µm thickness. DMEK was performed using an injector for intraocular lenses. All grafts were provided by the in-house cornea bank and prepared by the surgeons. We only used filtered room-air for the lamellar procedures. In phakic eyes, DMEK and DSAEK were always combined with cataract surgery as a triple procedure. This approach was chosen to mitigate the risk of ECD loss associated with performing cataract surgery on an eye after EK at a later stage. In contrast, for PK, combined cataract surgery was performed only when clinically indicated. The immediate postoperative treatment consisted of the administration of topical dexamethasone (Dexa EDO 1.3 mg/ml, Dr. Gerhard Mann GmbH or Monodex 1 mg/ml, Thea Pharma GmbH) five times daily, tapered over 5 months. For epithelial defects, dexpanthenol (Bepanthen 5%, Bayer HealthCare) and ofloxacin (Floxal 3 mg/g, Dr. Gerhard Mann GmbH) eye ointments were administered until reepithelialization was achieved. Following this initial period, patients received topical dexamethasone once daily as long-term therapy unless they showed signs of steroid-induced ocular hypertension.

Graft survival, endothelial cell density (ECD), rejection episodes, and best spectacle-corrected visual acuity (BSCVA) were retrospectively determined from medical records. Graft failure was defined as loss in function, requiring a repeat keratoplasty procedure. This was almost always due to corneal edema due to endothelial decompensation. The decision to perform a repeat keratoplasty was based on the clinical judgment of the treating ophthalmologist, considering factors such as visual acuity, corneal clarity, and patient preference. ECD was determined using a semiautomated cell counting method with a non-contact specular microscopy. Any differential diagnostic record of an endothelial rejection episode was considered a rejection in the statistics. Signs of endothelial rejection typically include new endothelial precipitates on the graft but not on the host cornea or otherwise unexplained graft edema. In case of suspected or confirmed rejection, the patient was intensively treated with topical dexamethasone.

We employed Kaplan–Meier survival analysis to evaluate graft survival, rejection risk, endothelial cell loss, and visual recovery over a 10-year period after DMEK, DSAEK, and PK. Multifactorial analyses were performed with the Cox proportional hazard model adjusting for the covariates triple-procedure, patient age, patient sex, donor age, organ culture duration, and preoperative ECD. All calculations were done using the software “R” (http://www.R-project.org, version 4.2.3). The alpha level was set to 0.05. We did not correct for multiple testing due to the descriptive nature of our study.

### Ethics approval and consent

This study was approved by the Ethics Committee of Albert-Ludwigs-Universität Freiburg (Antrag-Nr. EK-Freiburg: 23-1213-S1-retro). All methods were performed in accordance with relevant guidelines and regulations. For this retrospective study, the need for informed consent was waived by the Ethics Committee of Albert-Ludwigs-Universität Freiburg, as data were anonymized and retrieved from existing medical records.

## Results

**Fig. 1:**
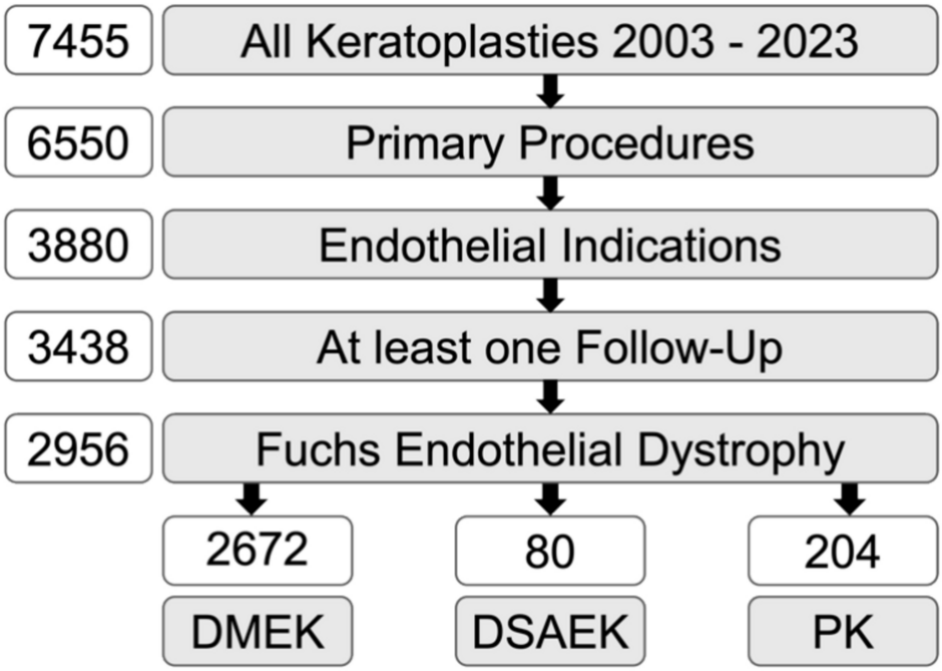
Flowchart of study inclusion criteria and resulting patient cohort.

Of the 7455 keratoplasties performed at the Eye Center between November 2003 and October 2023, only first-time keratoplasty eyes treated for Fuchs Endothelial Corneal Dystrophy with at least one follow-up visit were included in the study (Fig. 1). The final cohort consisted of 2956 first-time keratoplasties, with DMEK being the most common procedure (n = 2672), followed by PK (n = 204) and DSAEK (n = 80). The number of patients remaining in each group at ten years was 20 for DMEK, 9 for DSAEK, and 51 for PK. Eight surgeons performed these procedures. The median age of recipients across all groups was 71 years for DMEK and PK and 72 years for DSAEK. Many procedures, especially in the DMEK group (61%), were combined with phacoemulsification and intraocular lens implantation (triple procedures). Additional patient and donor characteristics are presented in Table 1.

**Table 1:** Baseline characteristics of transplant recipients, grafts, and donors. Values are presented as percentages or medians with interquartile ranges.

	DMEK	DSAEK	PK
n (Σ = 2956)	2672	80	204
Patient age in years	71 (64, 77)	72 (66, 77)	71 (65, 78)
Sex (female/male)	59% (n = 1582)/41%	52% (n = 42)/48%	60% (123)/40%
Triple procedure	61% (1621)	20% (16)	27% (55)
Follow-up in days	384 (96, 932)	1288 (337, 2549)	1829 (669, 3657)
Median donor age in years	71 (63, 79)	71 (60, 69)	73 (63, 79)
Organ culture duration in days	26 (22, 28)	20 (16, 25)	20 (16, 23)
Preoperative ECD	2336 (2190, 2482)	2336 (2190, 2555)	2409 (2263, 2555)
Postmortem time in days	0.98 (0.71, 1.58)	0.73 (0.56, 1.04)	1.01 (0.70, 1.50)
Rebubbling	19% (508)	3% (2)	–

Triple procedure refers to a combination of keratoplasty with phacoemulsification and intraocular lens implantation. *ECD* endothelial cell density.

### Graft survival

**Fig. 2:**
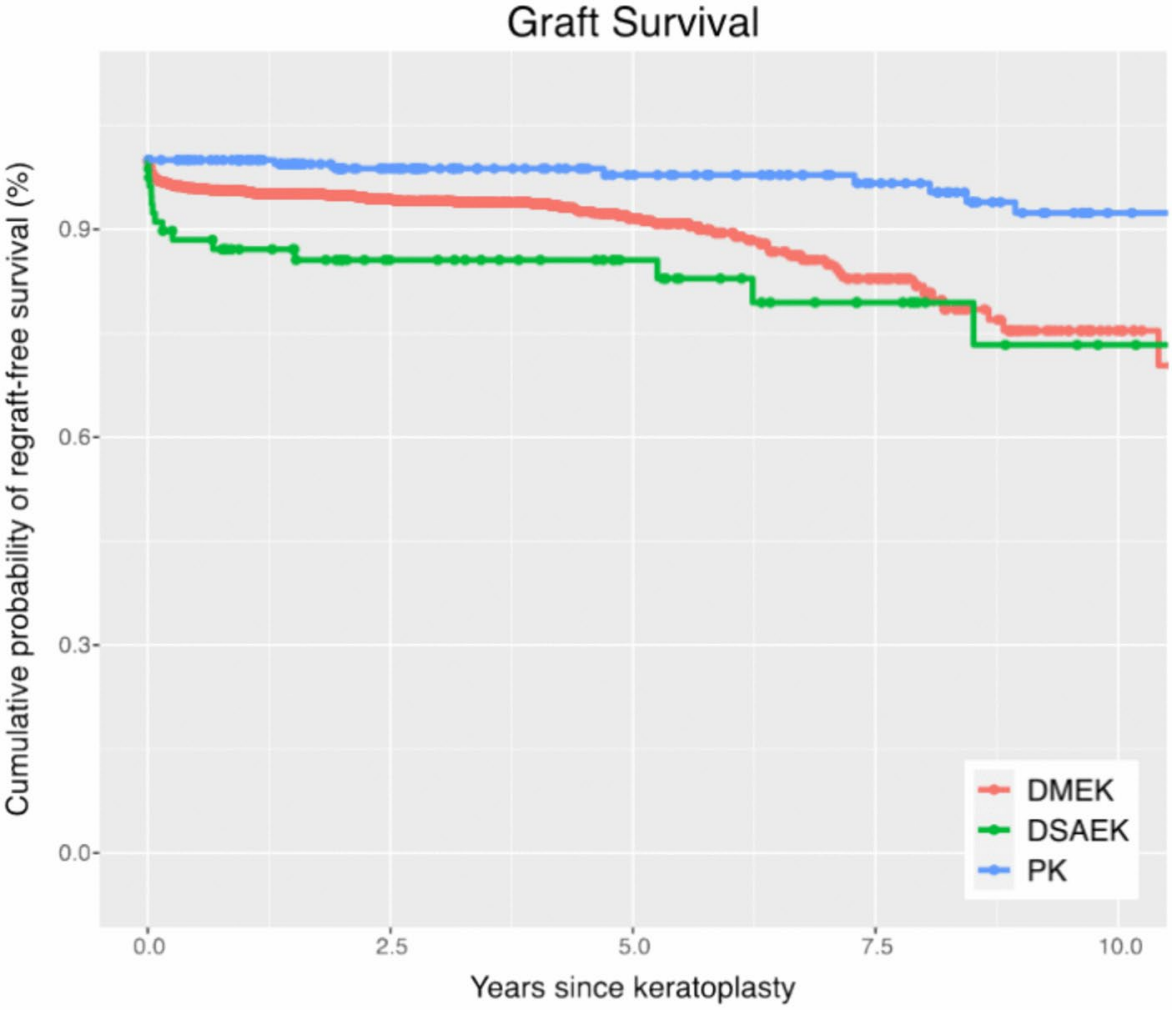
Graft survival: Kaplan–Meier cumulative probability of regraft-free survival within ten years after DMEK, DSAEK, and PK. The survival rate is higher after PK compared to DMEK and DSAEK. *DMEK* Descemet membrane endothelial keratoplasty, *DSAEK* Descemet stripping automated endothelial keratoplasty, *PK* penetrating keratoplasty.

**Fig. 3:**
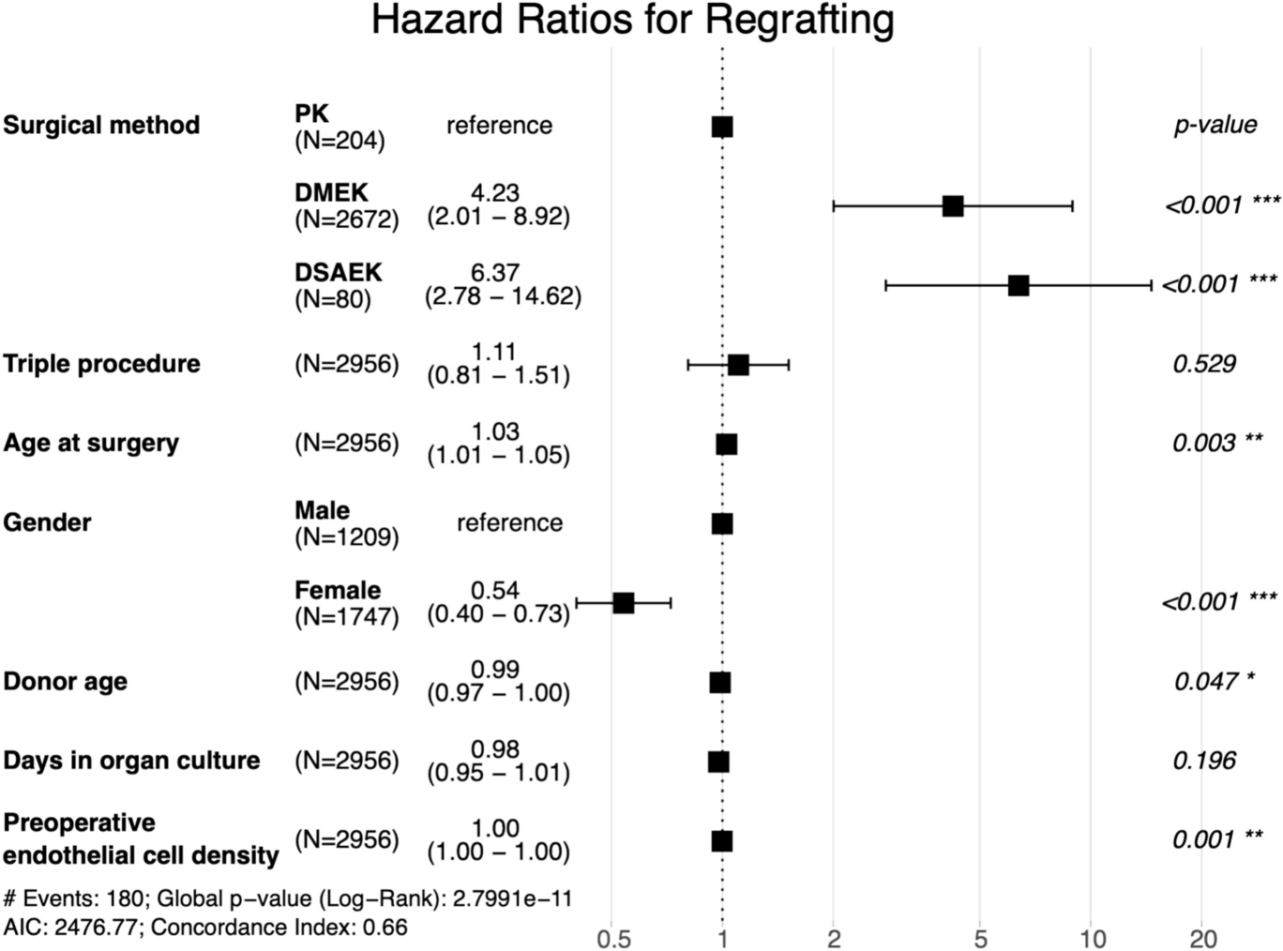
Hazard ratios for regrafting: Forest plot depicting adjusted hazard ratios with 95% confidence intervals for regrafting within ten years after PK, DMEK, and DSAEK. Hazard ratios are adjusted for clinical and demographic covariates, as listed on the left side of the plot. Triple procedure refers to a combination of keratoplasty with phacoemulsification and intraocular lens implantation. The probability of regrafting is higher after DMEK and DSAEK compared to PK. *DMEK* Descemet membrane endothelial keratoplasty, *DSAEK* Descemet stripping automated endothelial keratoplasty, *PK* penetrating keratoplasty.

PK demonstrates superior long-term graft survival compared to DMEK and DSAEK. The cumulative probability of graft survival postoperatively at 5 and 10 years was 92% and 75% for DMEK, 86% and 73% for DSAEK, and 98% and 92% for PK. PK showed a higher 10-year survival probability than the endothelial keratoplasty techniques DMEK and DSAEK (p < 0.001). (Table 2, Fig. 2 and 3).

**Table 2:** Kaplan–Meier cumulative probabilities for graft survival, ECD above 1000 cells/mm^2^, graft rejection, and BSCVA ≥ 6/12 five and ten years after DMEK, DSAEK, and PK.

	Years since KP	Kaplan–Meier cumulative probability			p-values for hazard ratios	
		DMEK	DSAEK	PK	DMEK/PK	DSAEK/PK
Graft survival	5	92% (90, 94)	86% (78, 94)	98% (95, 100)		
	10	75% (69, 83)	73% (60, 91)	92% (87, 98)	< 0.001	< 0.001
ECD > 1000 cells/mm^2^	5	25% (22,29)	34% (22, 52)	50% (42, 60)		
	10	3% (1, 7)	8% (2, 40)	18% (11, 29)	< 0.001	< 0.001
No rejection episode	5	97% (95, 98)	85% (76, 94)	88% (82, 94)		
	10	90% (84, 97)	81% (71, 93)	87% (81, 93)	0.003	0.186
BSCVA < 6/12 SNELLEN	5	7% (5, 8)	17% (9, 32)	37% (30, 46)		
	10	1% (0, 5)	-	22% (15, 31)	< 0.001	< 0.001

The p-values for hazard ratios from a multivariate Cox proportional hazards regression analysis are provided for comparisons between DMEK and PK, as well as DSAEK and PK. *KP* Keratoplasty, *DMEK* Descemet membrane endothelial keratoplasty, *DSAEK* Descemet stripping automated endothelial keratoplasty, *PK* penetrating keratoplasty, *ECD* endothelial cell density *BSCVA* best spectacle-corrected visual acuity.

### Endothelial cell density loss

**Fig. 4:**
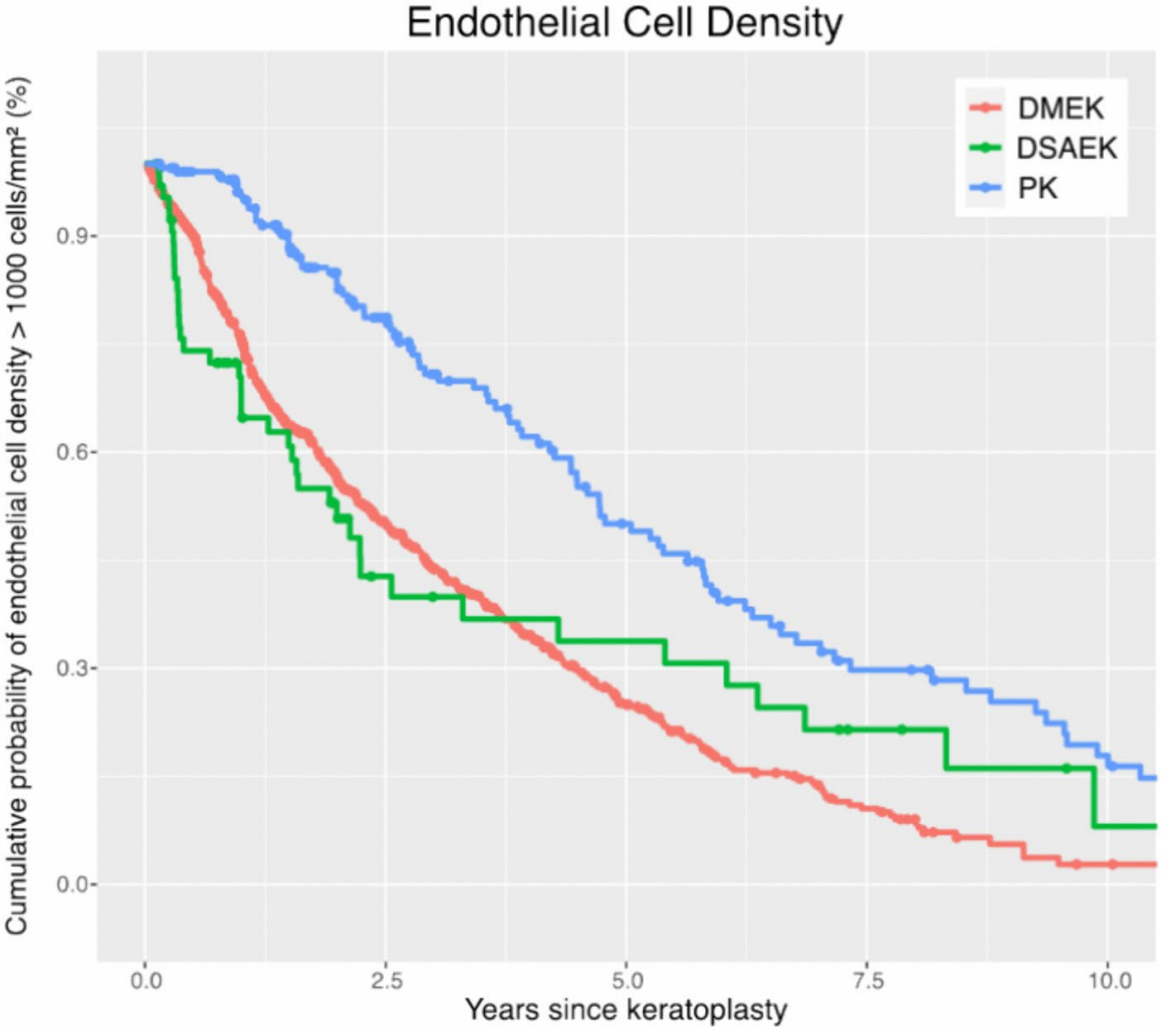
Endothelial cell density loss: Kaplan–Meier cumulative probability of ECD dropping below 1000 cells/mm^2^ within ten years after DMEK, DSAEK, and PK. ECD drops faster following DMEK and DSAEK compared to PK. *ECD* Endothelial cell density, *DMEK* Descemet membrane endothelial keratoplasty, *DSAEK* Descemet stripping automated endothelial keratoplasty, *PK* penetrating keratoplasty.

**Fig. 5:**
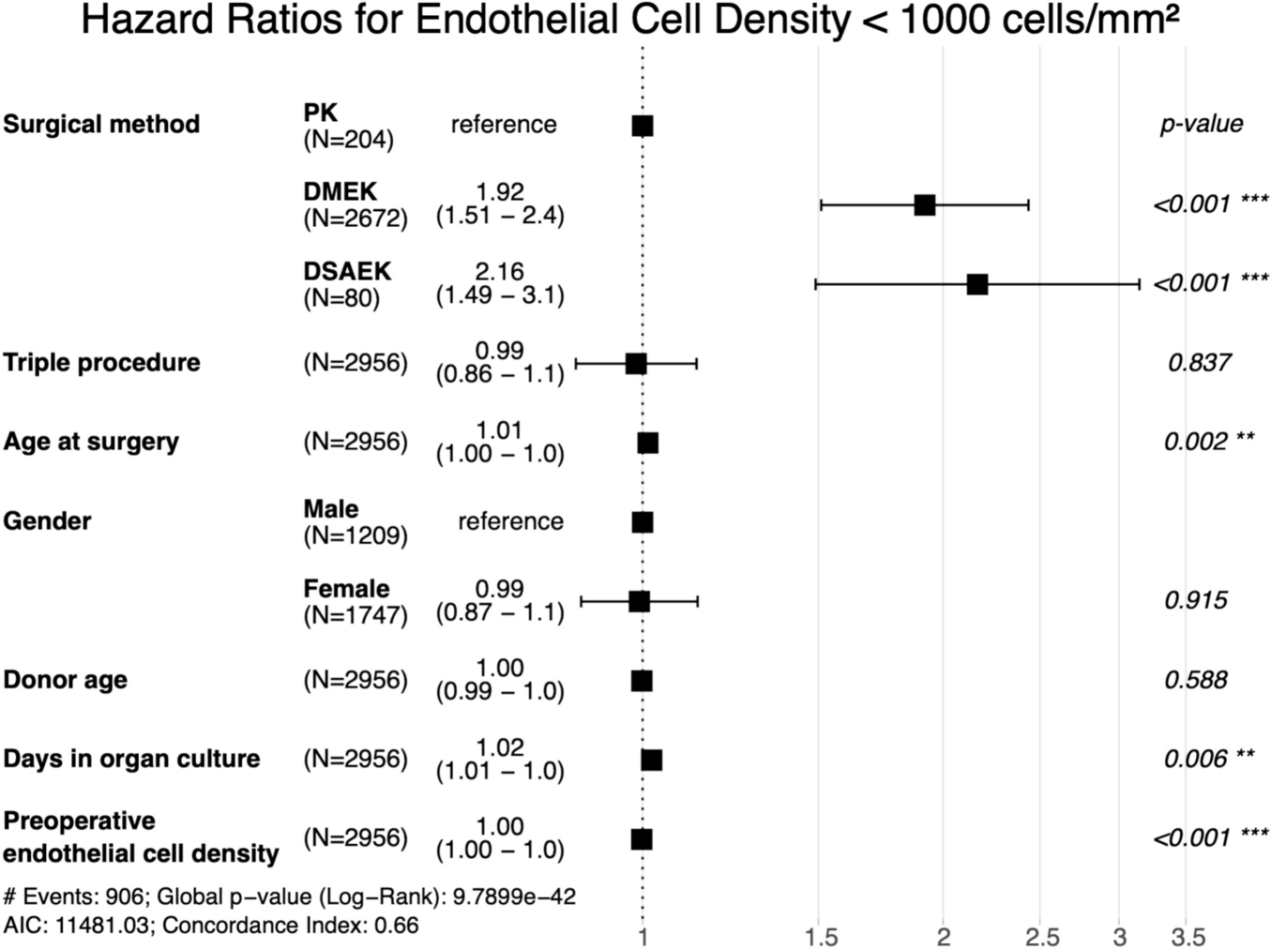
Hazard Ratios for Endothelial Cell Density < 1000 cells/mm^2^: Forest plot depicting adjusted hazard ratios with 95% confidence intervals for endothelial cell density dropping below 1000 cells/mm^2^ within ten years after PK, DMEK, and DSAEK. Hazard ratios are adjusted for clinical and demographic covariates, as listed on the left side of the plot. Triple procedure refers to a combination of keratoplasty with phacoemulsification and intraocular lens implantation. The risk of endothelial cell density being lower than 1000 cells/mm^2^ is higher after DMEK and DSAEK compared to PK. *DMEK* Descemet membrane endothelial keratoplasty, *DSAEK* Descemet stripping automated endothelial keratoplasty, *PK* penetrating keratoplasty.

Postoperative ECD decreases faster after DMEK and DSAEK than after PK (p < 0.001) (Table 1). Five and ten years after surgery, the cumulative probability of maintaining an ECD above 1000 cells/mm^2^ was 25% and 3% for DMEK, 34% and 8% for DSAEK, and 50% and 18% for PK, respectively (Figs. 4 and 5, Table 2). In the long term, there appears to be a trend towards convergence in the probability of maintaining an ECD above 1000 cells/mm^2^ between the different techniques.

### Graft rejection

**Fig. 6:**
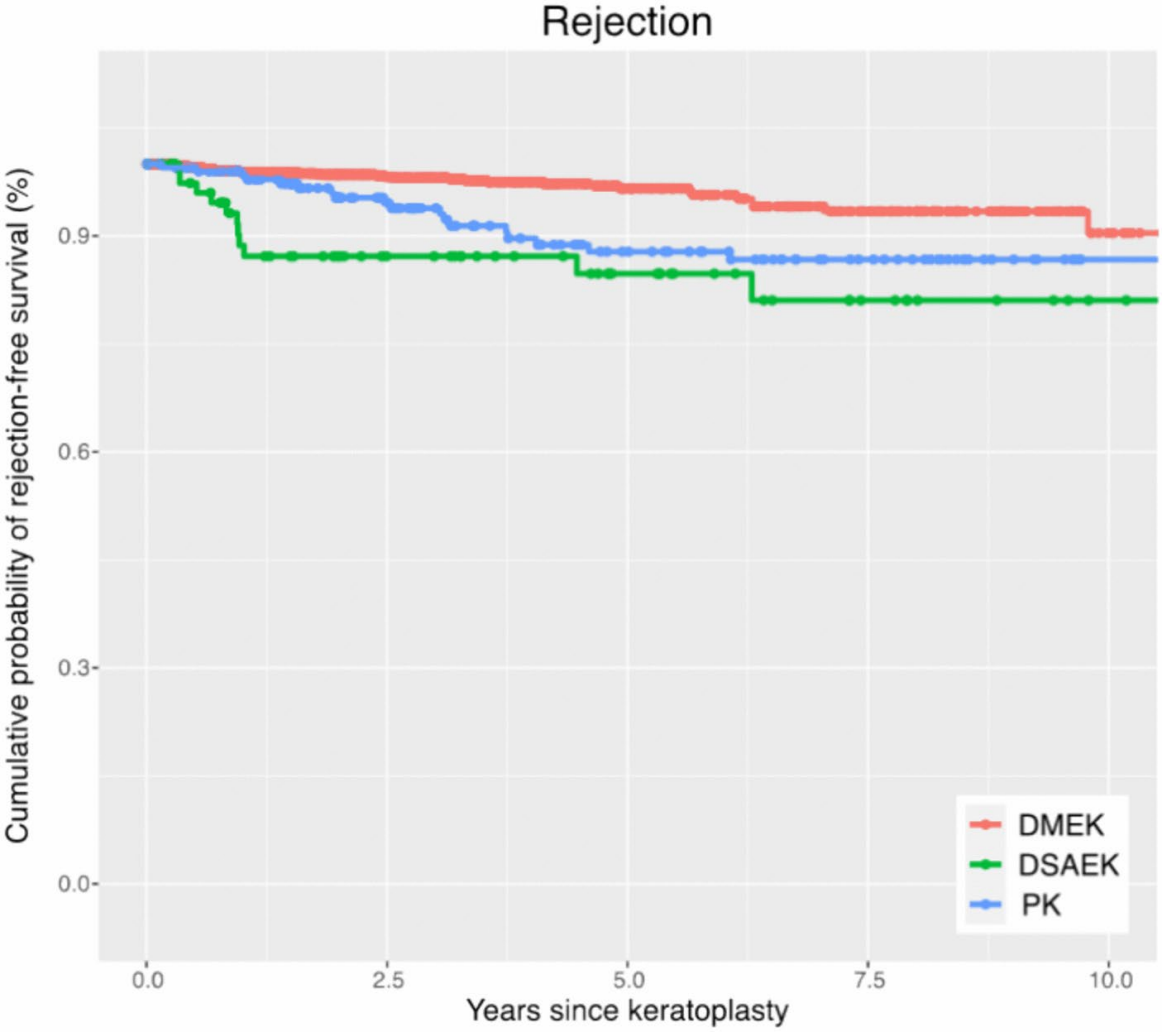
Rejection-free survival: Kaplan–Meier cumulative probability of rejection-free survival within ten years after DMEK, DSAEK, and PK. Higher rejection rates are observed after PK compared to DMEK. *DMEK* Descemet membrane endothelial keratoplasty, *DSAEK* Descemet stripping automated endothelial keratoplasty, *PK* penetrating keratoplasty.

**Fig. 7:**
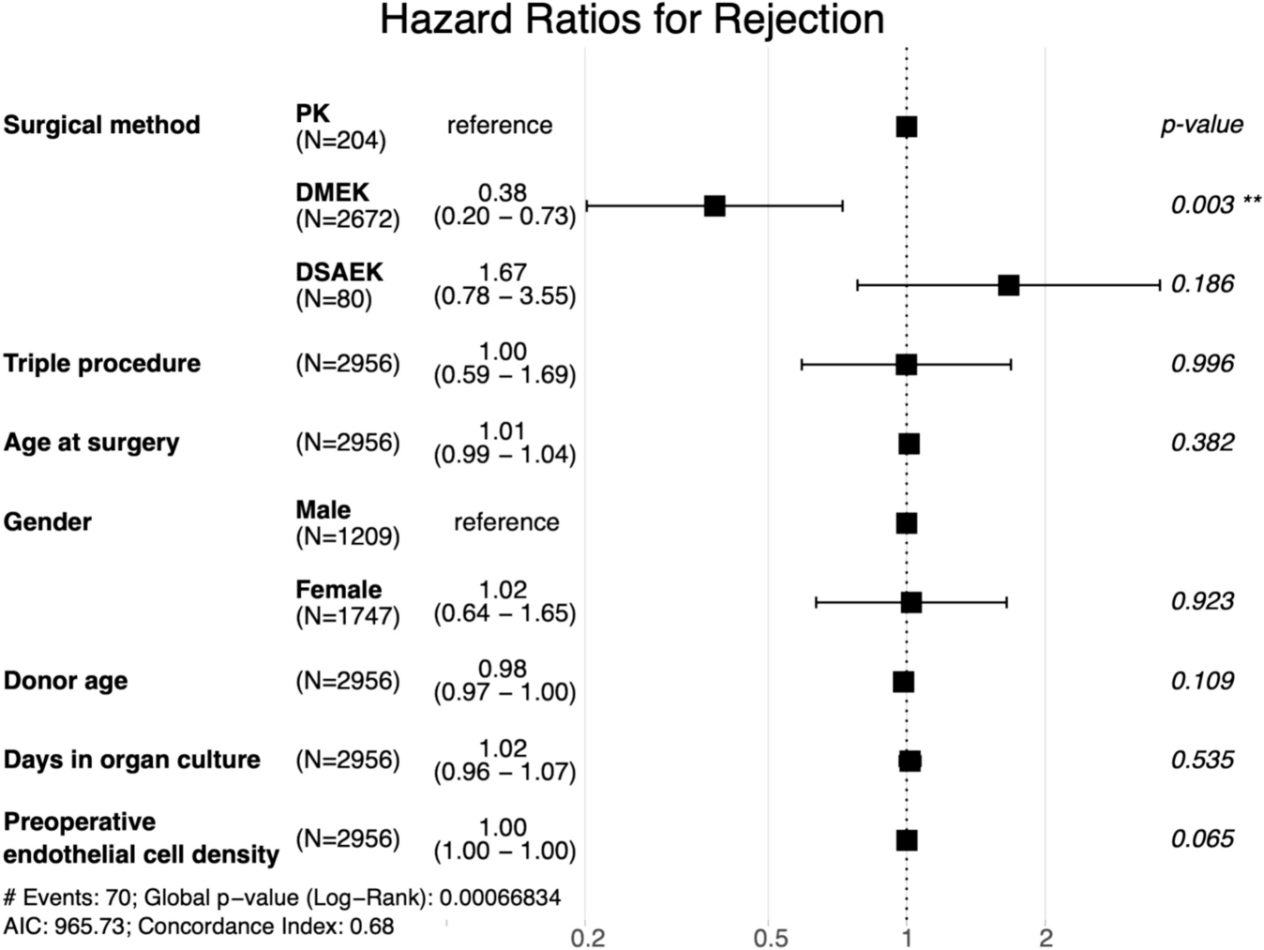
Hazard ratios for rejection: Forest plot depicting adjusted hazard ratios with 95% confidence intervals for rejection within ten years after PK, DMEK, and DSAEK. Hazard ratios are adjusted for clinical and demographic covariates, as listed on the left side of the plot. Triple procedure refers to a combination of keratoplasty with phacoemulsification and intraocular lens implantation. The risk of rejection is lower after DMEK compared to PK. *DMEK* Descemet membrane endothelial keratoplasty, *DSAEK* Descemet stripping automated endothelial keratoplasty, *PK* penetrating keratoplasty.

Within ten years postoperatively, the highest cumulative probability of suspected graft rejection was after DSAEK (19%), followed by PK (13%) and DMEK (10%). After DMEK, there occurred significantly less suspected graft rejection than after PK (p = 0.003), while we saw no significant difference after DSAEK and PK (p = 0.186) (Figs. 6 and 7, Table 2).

### Visual acuity

**Fig. 8:**
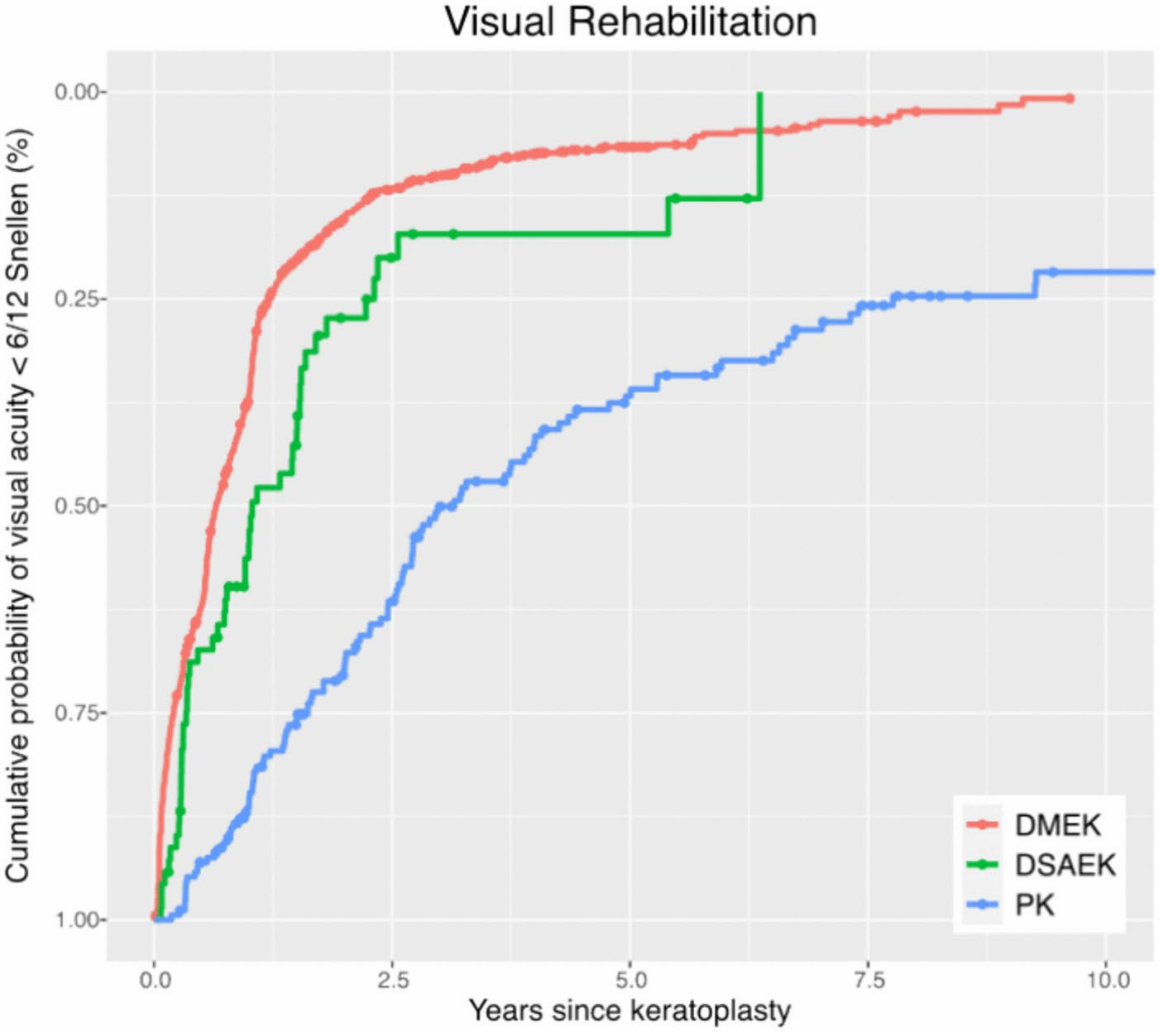
Visual acuity rehabilitation: Kaplan–Meier cumulative probability of visual rehabilitation following keratoplasty (DMEK, DSAEK, and PK). BSCVA of 6/12 is achieved faster following DMEK and DSAEK compared to PK. *DMEK* Descemet membrane endothelial keratoplasty, *DSAEK* Descemet stripping automated endothelial keratoplasty, *PK* penetrating keratoplasty.

**Fig. 9:**
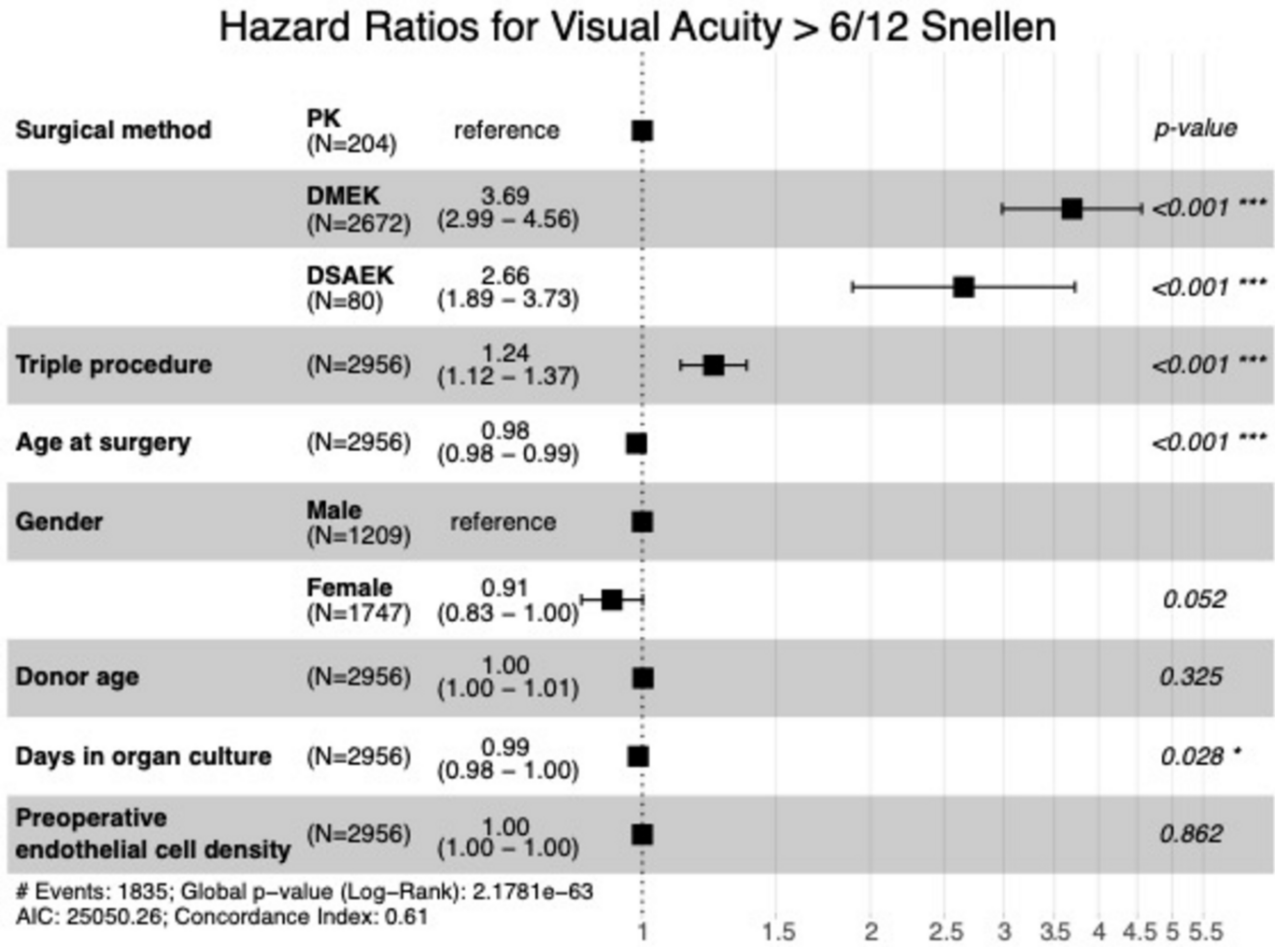
Hazard Ratios for Visual Acuity > 6/12 Snellen: Forest plot depicting adjusted hazard ratios with 95% confidence intervals for reaching a vision better than BSCVA 6/12 Snellen within ten years after PK, DMEK, and DSAEK. Hazard ratios are adjusted for clinical and demographic covariates, as listed on the left side of the plot. Triple procedure refers to a combination of keratoplasty with phacoemulsification and intraocular lens implantation. The probability of reaching a visual acuity above 6/12 Snellen is higher after DMEK and DSAEK compared to PK. *DMEK* Descemet membrane endothelial keratoplasty, *DSAEK* Descemet stripping automated endothelial keratoplasty, *PK* penetrating keratoplasty.

All eyes, regardless of any vision-impairing comorbidities, were included. Visual recovery post-transplantation showed a faster improvement in eyes undergoing endothelial keratoplasty compared to those receiving PK (p < 0.001). The cumulative probability of having a BSCVA better than SNELLEN 6/12 five and ten years after the surgery was 93% and 99% for DMEK, 63% and 88% for PK, and 83% after five years for DSAEK. The median time until a BSCVA ≥ 0.5 SNELLEN was reached was 7.8 (95% CI 7.3/8.6) months for DMEK, 12.4 (95%CI 9.2/18.4) months for DSAEK, and 37.9 (95% CI 31.6/48.8) months for PK. (Fig. 8 and 9, Table 2).

## Discussion

Our study significantly contributes to the existing literature on corneal transplantation by comparing long-term outcomes of endothelial keratoplasty (DMEK, DSAEK) and PK over a ten-year period in a real-world clinical setting, considering learning curves and ocular comorbidities. The data originates from one of Germany’s largest keratoplasty centers, providing insights from a high-volume context. Notably, DMEK adoption is particularly high in Germany compared to other regions, while DSAEK was only performed between January 2009 and May 2013 at our eye center, potentially affecting the generalizability of our findings to countries with different practices. Historically, PK represented the primary surgical intervention for FED, but was progressively replaced by DMEK, which is suspected to offer less invasive procedures and superior visual outcomes. The introduction of DMEK might have lowered the threshold for performing keratoplasty, potentially explaining the disparity in cohort sizes between PK and DMEK.

However, this high acceptance of DMEK suggests that our results represent a best-case scenario for DMEK at a single high-volume center, offering a robust basis for comparing DMEK and PK outcomes.

Given that DMEK has been the predominant keratoplasty technique at the eye center in recent years, the median follow-up period for this group is shorter. This imbalance is accounted for in the Kaplan–Meier survival analysis.

### Graft survival

Contrary to initial expectations, PK demonstrated superior long-term survival compared to endothelial keratoplasty. This finding aligns with Australian^11,13,14^ and United Kingdom (only DSEK and DSAEK)^10^ registry studies but contrasts with some single-center reports or shorter-term studies^7–9^. Our 10-year graft survival rates (75% for DMEK, 73% for DSAEK, and 92% for PK) highlight a significant challenge for endothelial keratoplasty that warrants further investigation. It should be considered that the higher surgical trauma and higher risk of subsequent immune reactions associated with repeat PK may influence the willingness of surgeons and patients to undergo repeat keratoplasty, possibly skewing our survival statistics.

### Endothelial cell density loss

Maintaining sufficient ECD is crucial for corneal clarity and, thus, for long-term graft function. A minimum ECD of 400 to 500 cells/mm^2^ is required for endothelial functionality^5^. Our study demonstrated a significantly faster ECD loss after endothelial keratoplasty compared to PK. At ten years post-operatively, only 3% of DMEK grafts and 8% of DSAEK grafts maintained an ECD above 1000 cells/mm^2^, contrasting with 18% for PK. This accelerated ECD loss in endothelial keratoplasty likely contributes to its lower ten-year graft survival rates.

The mechanisms underlying this accelerated endothelial cell loss are not fully understood, but potential contributing factors include surgical trauma, graft detachment, immune reactions, and the choice of tamponade agent. Endothelial immune reactions appear to be associated with a higher risk of endothelial cell loss than stromal immune reactions^15^. Research in feline models has suggested that sulfur hexafluoride (SF6) gas causes more significant endothelial cell loss and inflammation than air^16^. However, a fellow eye comparison in humans did not reproduce these findings and even recommended SF6 due to lower graft detachment rates^17^. At the Eye center, only air was used for primary keratoplasties to minimize potential ECD loss risk. While donor-related factors such as increased postmortem time and advanced donor age are also thought to negatively influence endothelial cell loss in PK, these factors are unlikely to contribute to the observed differences in our study, as donor age was comparable across all groups, and postmortem time was comparable between DMEK and PK and even shorter for DSAEK (see Table 1)^18^. An important consideration in our analysis is the systematic use of triple procedures, particularly in the DMEK group, where 61% of cases involved simultaneous phacoemulsification and intraocular lens implantation. Notably, cataract surgery was performed prior to graft implantation, allowing the triple procedure to minimize the need for subsequent surgeries while being unlikely to affect long-term endothelial cell density (ECD) dynamics. Additionally, graft repositioning procedures, known as rebubbling, which are performed in cases of graft detachment, could influence ECD loss, i.e. in DMEK where the rebubbling-rate is nearly 20% (Table 1).

The literature on long-term ECD loss shows inconsistent results. Some studies report higher ECD loss after PK^7,8^, while others are consistent with our findings of greater loss after DMEK and DSAEK^9,12^. Interestingly, the probability of reaching the 1000 cells/mm^2^ threshold appears to converge among the three surgical techniques in the long term. Equalization of endothelial cell counts after 10 years has also been described previously for DSAEK and PK^19^. The rapid loss of ECD in endothelial keratoplasty represents a critical finding that may explain the lower long-term graft survival rates observed.

Emerging therapies, such as Rho-associated kinase (ROCK) inhibitors, hold potential to protect against apoptosis and promote endothelial cel proliferation and, therefore, represent a promising avenue for further research to optimize keratoplasty outcomes^20^.

### Graft rejection

Our study confirms the immunological advantages of DMEK, with the lowest rate of suspected graft rejection (10% at 10 years) compared to DSAEK (19%) and PK (13%)^9,12,21,22^. This supports the concept of immunological privilege in DMEK, possibly due to the transplantation of less foreign tissue. The absence of donor stroma and epithelium in DMEK may reduce the antigenic load and subsequent immune response^21,23,24^. Furthermore, the sutures required in PK, which are absent in endothelial keratoplasty, may promote vascular ingrowth and thus induce rejection^24^. One factor known to reduce the incidence of endothelial immune responses is an organ culture duration of more than 21 days, which was the case for DMEK in our study. This potential confounder was considered in the multivariate Cox proportional hazard regression analysis, which nevertheless revealed a significantly lower graft rejection rate for DMEK^25^.

The unexpectedly high rejection rates after DSAEK, though not significantly different from PK, may be attributed to the presence of donor stromal tissue in the graft. The exposure of this donor stroma, potentially containing antigen-presenting cells of the donor to the anterior chamber, could induce graft rejection by compromising the cornea’s immune privilege. The limited number of DSAEK cases at our center after its early replacement by DMEK could also introduce a bias in this observation.

### Visual acuity

Visual acuity outcomes following keratoplasty significantly contribute to patient satisfaction and quality of life improvements^26^. In our analysis, we investigated the threshold BSCVA 6/12 Snellen, the minimum for a driving permit in Germany^27^. Both DMEK and DSAEK demonstrated superior visual outcomes compared to PK, with significantly faster visual rehabilitation. The threshold BSCVA ≥ 6/12 Snellen was reached 7.8 months after DMEK, 12,4 months after DSAEK and 37,9 months after PK. At 10 years, 99% of DMEK and 88% of PK patients achieved a BSCVA of ≥ 6/12 Snellen. The objective of our analysis was to investigate outcomes in a real-world clinical setting. Therefore, we intentionally included all patients without stratifying by ocular comorbidities. While we recognize that ocular comorbidities may affect visual outcomes, this limitation is likely consistent across all three cohorts. As age at the time of surgery was similar among the groups, it is likely that the proportion of eyes with prior surgeries is comparable. Our results align with previous studies reporting improved visual outcomes and reduced refractive error with endothelial keratoplasty techniques^12,28,29^. While PK demonstrates slower visual recovery, it’s important to note that most patients achieve good long-term visual results. The actual visual acuity achieved with PK is likely even higher than reported, as routine clinical testing is often performed without rigid contact lenses, which are mostly necessary to achieve optimal visual acuity after penetrating keratoplasty. However, the induced astigmatism and the need for contact lenses and suture removal reduce PK’s practicability.

The superior visual outcomes of endothelial keratoplasties, along with lower rejection rates—particularly for DMEK—and greater practicability (no sutures, no need for contact lenses), support their consideration as primary treatment options for endothelial diseases, despite the challenges in long-term graft survival and increased ECD loss. Additionally, DMEK is easily and less traumatically repeatable in case of late endothelial failure. Future studies should further investigate the feasibility and consequences of repeat DMEK, considering factors such as cumulative endothelial cell loss and possible immune sensitization.

## Limitations

While our study provides valuable insights, several limitations should be considered when interpreting the results. Due to the retrospective design, there is a potential for loss of follow-up, which we addressed using statistical methods that cope with censored data. Our reliance on real-world data may include diagnosis inaccuracies and patient comorbidities. However, these limitations should be similar across all three groups and thus not induce any major bias.

The Eye Center’s isolated location between the Black Forest and the borders to Switzerland and France suggests that most major events were likely recorded, as there is no alternative center within easy reach for most patients. This geographical factor may have inadvertently improved our follow-up rates and data completeness.

The significant difference in the number of procedures among the groups (DMEK, DSAEK, PK) represents an imbalance in sample sizes, potentially impacting the statistical power and the comparability of outcomes. To mitigate this, we consecutively included all eligible eyes and pre-defined our outcome measures before data analysis. However, we acknowledge that some limitations in comparability may remain due to the inherent differences in group sizes.

In addition, it should be noted that the DMEK and DSAEK statistics may include a stronger learning curve, as these techniques were just emerging and evolving in contrast to the established PK procedures during our study period.

A further limitation is that surgeon-specific factors were not controlled for in the analysis. However, there were only eight surgeons, performing a similarly high volume of cases and adhering to standardized protocols. Although the analysis did not control for surgeon-specific factors, the small cohort of eight surgeons, who performed a comparable high volume of cases and followed standardized protocols, mitigates potential variability.

The inclusion of all eyes on which a first keratoplasty was performed leads to a potential patient-specific bias, which, however, should be low due to the large case number and the fact that FED usually affects both eyes.

It is important to note that during the study period, our eye center did not utilize ultra- or nano-thin DSAEK grafts, as these techniques were still evolving. Consequently, our DSAEK results represent conventional graft techniques prevalent at the time and may not reflect the potential improvements or difficulties associated with thinner grafts.

## Conclusion

Key findings on ten-year outcomes:

- Superior graft survival in PK compared to DMEK and DSAEK
- Lower rejection rates in DMEK compared to PK
- Slower endothelial cell loss in PK compared to DMEK and DSAEK
- Faster visual improvement in DMEK and DSAEK compared to PK

Our study shows that while long-term graft survival for endothelial keratoplasty procedures is limited compared to PK—potentially due to faster ECD loss—endothelial keratoplasty techniques offer significant advantages regarding visual outcomes and rejection rates, particularly for DMEK. The potential for repeatability in endothelial keratoplasty further supports its consideration as a primary treatment option for endothelial diseases.

Focusing on procedures performed in a high-volume center, our study provides valuable insights into the best-practice outcomes achievable with different keratoplasty techniques. The real-life approach, which includes all comorbidities and uncertainties, allows an accurate assessment of the true potential and limitations of each surgical technique, which is important for patient information, clinical decision-making, and future research directions in corneal transplantation.

Despite the limitations of a single-center retrospective study, our extensive data collection, large sample size, especially for DMEK procedures, long follow-up period, and high frequency of procedures performed at our center contribute significantly to the strength and relevance of our findings in the field of corneal transplantation.

## Data Availability

The datasets generated and analyzed during the current study are available from the corresponding author upon reasonable request. Access to raw data may require a data-sharing agreement in compliance with institutional and data protection regulations.

## Declarations

### Generative AI and AI-assisted technologies in the writing process

Grammarly v1.72.3.0, DeepL, GPT-4o, and Claude 3.5 Sonnet were used for linguistic improvements. The authors carefully checked the text suggestions for accuracy and made corrections where necessary. They take full responsibility for the publication’s content.
